# Capillary leakage in post-cardiac arrest survivors during therapeutic hypothermia - a prospective, randomised study

**DOI:** 10.1186/1757-7241-18-29

**Published:** 2010-05-25

**Authors:** Bård E Heradstveit, Anne Berit Guttormsen, Jørund Langørgen, Stig-Morten Hammersborg, Tore Wentzel-Larsen, Rune Fanebust, Elna-Marie Larsson, Jon-Kenneth Heltne

**Affiliations:** 1 Department of Anaesthesia and Intensive Care, Haukeland University Hospital, Bergen, Norway; 2 Department of Surgical Sciences, University of Bergen, Bergen, Norway; 3 Medical Intensive Care Unit, Department of Heart Disease, Haukeland University Hospital, Bergen, Norway; 4 Centre for Clinical Research, Haukeland University Hospital, Bergen, Norway; 5 Department of Radiology, Uppsala University Hospital, Uppsala, Sweden; 6 Department of Medical Sciences, University of Bergen, Bergen, Norway

## Abstract

**Background:**

Fluids are often given liberally after the return of spontaneous circulation. However, the optimal fluid regimen in survivors of cardiac arrest is unknown. Recent studies indicate an increased fluid requirement in post-cardiac arrest patients. During hypothermia, animal studies report extravasation in several organs, including the brain. We investigated two fluid strategies to determine whether the choice of fluid would influence fluid requirements, capillary leakage and oedema formation.

**Methods:**

19 survivors with witnessed cardiac arrest of primary cardiac origin were allocated to either 7.2% hypertonic saline with 6% poly (O-2-hydroxyethyl) starch solution (HH) or standard fluid therapy (Ringer's Acetate and saline 9 mg/ml) (control). The patients were treated with the randomised fluid immediately after admission and continued for 24 hours of therapeutic hypothermia.

**Results:**

During the first 24 hours, the HH patients required significantly less i.v. fluid than the control patients (4750 ml versus 8010 ml, p = 0.019) with comparable use of vasopressors. Systemic vascular resistance was significantly reduced from 0 to 24 hours (p = 0.014), with no difference between the groups. Colloid osmotic pressure (COP) in serum and interstitial fluid (p < 0.001 and p = 0.014 respectively) decreased as a function of time in both groups, with a more pronounced reduction in interstitial COP in the crystalloid group. Magnetic resonance imaging of the brain did not reveal vasogenic oedema.

**Conclusions:**

Post-cardiac arrest patients have high fluid requirements during therapeutic hypothermia, probably due to increased extravasation. The use of HH reduced the fluid requirement significantly. However, the lack of brain oedema in both groups suggests no superior fluid regimen. Cardiac index was significantly improved in the group treated with crystalloids. Although we do not associate HH with the renal failures that developed, caution should be taken when using hypertonic starch solutions in these patients.

**Trial registration:**

NCT00347477.

## Background

Few studies have described fluid requirements in cardiac arrest patients [1-3], but fluid infusion after ROSC is increasingly debated [4]. During hypothermia, animal studies report extravasation in several organs, including the brain [5,6]. Whether capillary leakage is present in man during therapeutic hypothermia, is not documented. This is of clinical interest, as oedema formation in a vulnerable OHCA-brain is considered harmful. Furthermore, this is underlined by the similarity between post-resuscitation syndrome and sepsis [7,8]. Septic patients are known to have high fluid requirements, and outcome is improved by goal-directed fluid therapy [9]. Encouraged by the low cardiac output after cardiac arrest [1], fluid load would appear to be worth attempting. In addition, the induction of hypothermia by large volumes of cold intravenous infusions has gained in popularity [10].

The application of a hypertonic colloid during cardiopulmonary bypass has been shown to reduce fluid overload [11,12]. Colloids tend to cause less tissue oedema than crystalloids [13] and, as regards inflammatory-related leakages, hydroxyethyl starch could have an 'occlusive' effect on damaged capillaries, subsequently limiting extravasation [14]. Furthermore, hypertonic solutions recruit fluid from the intracellular space to the capillaries, and, during CPR in an animal model, these solutions increased myocardial blood flow and the survival rate [15].

The aim of the study was to determine whether a capillary leakage was present in OHCA survivors during therapeutic hypothermia. We compared two fluid regimens and studied the impact on capillary leakage. The intervention group received an additional 500 ml of 7.2% hypertonic saline with 6% poly (O-2-hydroxyethyl) starch solution during the first 24 hours, and was compared with standard therapy. The primary endpoint was the amount of fluid administered during the first 24 hours. The secondary endpoint was the magnitude of capillary leakage as a surrogate marker for oedema formation.

## Methods

### Ethics

The study was approved by the Regional Committees for Medical Research Ethics, the Data Inspectorate, the Directorate for Health and Social Affairs and the Norwegian Medicines Agency. Deferred consent was used, and the patients' families were entitled to withdraw the patients at any time. All patients included were informed about the study when they were able to receive the information and signed a written informed consent form.

### Study population and environment

The study was performed on 19 patients with witnessed out-of-hospital cardiac arrest (OHCA) and carried out between September 2005 and March 2007 at Haukeland University Hospital (Bergen, Norway), an 1,100-bed hospital serving 600,000 people. All inclusion/exclusion criteria are presented in Table 1. The fluid intervention was initiated immediately after admission to the emergency room and continued for the first 24 hours.

**Table 1:** Criteria for inclusion.

Inclusion criteria	Exclusion criteria
• Witnessed cardiac arrest with a probable cardiac cause. (Ventricular fibrillation, tachycardia, asystole and pulseless electrical activity)	• Terminal illness, strongly in need of nursing
• Advanced medical life support within 15 minutes	• Primary coagulopathy
• Return of spontaneous circulation within 60 minutes	• Prehospital fluid load >2000 ml
• Comatose when admitted to the hospital, (Glasgow Coma Score 3)	
• Age 18-80 years	

### Treatment protocol

On admission, the patients were allocated by means of stratified randomisation to one of two fluid regimens administered via infusion pumps: Ringer's Acetate and saline 9 mg/ml (control), or hypertonic colloid,7.2% NaCl with 6% Hydroxyethyl starch 200/0.5 (HyperHAES^® ^Fresenius Kabi, Germany) (HH). Fluid was administered to achieve the treatment goals listed in Table 2. HH was limited to 500 ml per 24 hours (20 ml/hr). Further needs for fluid in the HH group were met by Ringer's Acetate/saline 9 mg/ml. The control group received Ringer's Acetate and saline 9 mg/ml by turn during the observation period, in accordance with the standard treatment in the medical intensive care unit (MICU).

**Table 2:** Treatment goals.

Parameter	**Treatment goal**s
Blood pressure	MAP > 60 mmHg
Heart rate	60-100 min^-1^
Central venous pressure	8-12 mmHg
Temperature	33°C
Blood gases	pH 7.35-7.45
	pO_2 _10-12 kPa
	pCO_2 _5-6 kPa
Blood glucose	5-8 mmol/l
Electrolytes	Within normal range
Hb	>9 g/dl
Diuresis	>1 ml/kg/hrs

### Coronary intervention

Patients with ST elevation, a new left bundle branch block or cardiogenic shock were referred immediately for coronary angiography and subsequent percutaneous coronary intervention (PCI).

### Magnetic resonance imaging

Before admission to the MICU, after cardiac intervention and if the patient did not have an intra-aortic-balloon pump (IABP), magnetic resonance imaging (MRI) of the brain was planned (1.5 Tesla, conventional morphological and diffusion sequences). Repeated MRI was scheduled after 24 and 96 hours.

### Intensive care treatment and monitoring

Cardiac arrest data were recorded according to the Utstein style [16]. In the MICU, monitoring was performed (IntelliVue, Philips, Eindhoven, the Netherlands) with continuous ECG, arterial pressure and continuous cardiac output registration (PiCCO^®^, Pulsion Medical System AG, Germany). Fluid balance was measured as the total amount of fluid administered intravenously and enterally in relation to output measured by hourly diuresis and 24-hour faecal loss. Systemic vascular resistance (SVR) was calculated 0, 8, 16 and 24 hours after admission to the MICU. Vasopressors (dopamine, noradrenaline, and adrenaline) were administered if the mean arterial blood pressure was <60 mmHg and the fluid load proved ineffective, guided by PiCCO measurements. Dopamine was replaced with noradrenaline if tachycardia occurred (>100 beats min^-1^) or if dopamine failed to achieve the required blood pressure. Sedatives (midazolam, alfentanil) were administered to achieve a motor activity assessment score of 0 (MAAS). If necessary, vecuronium was administered to prevent shivering. Ventilation was provided by Evita XL (Dräger Medical, Lübeck, Germany), using a bi-positive airway pressure mode. Cooling was initiated outside the hospital for all patients who had return of spontaneous circulation (ROSC) and remained unconscious. At the scene, cooling was performed using icepacks placed on the neck, armpits and groin. A Coolgard catheter (Alsius, California, USA) was installed in the right femoral vein in the PCI lab and activated in the MICU, cooling the patient at a rate of 1°C per hour. The target temperature was set at 33°C and measured in the urine bladder. After 24 hours of cooling, rewarming at a rate of 0.5°C per hour was stopped at 35.0°C.

### Blood samples and sampling of interstitial fluid

Blood samples were taken from the artery line after 0, 8, 16 and 24 hours, and analysed at the Laboratory of Clinical Biochemistry at Haukeland University Hospital. Colloid osmotic pressure (COP) was measured at 0, 8, 16 and 24 hours in serum and in interstitial fluid that was sampled using the wick method, installed for 60 minutes [17-20]. A sterile, multi-filament nylon wick was soaked in Ringer AC. Using a sterile technique and a needle, the wick was placed subcutaneously in the midaxillary line. Three wicks were installed at intervals of 3 cm and covered by plastic film (Tegaderm, 3M Inc., Canada), to prevent evaporation. COP was measured by means of a transducer (Gould-Statham, Spectramed, USA), recorded and amplified with an EasyGraph 240 (Gould Inc., USA).

### Statistical analysis

The randomisation was stratified with respect to initial heart rhythm. Numbered envelopes were distributed from the MICU and opened when the physician in the emergency room enrolled a patient, filling in the inclusion criteria. The allocation was generated by the authors. The sample size was determined by power calculations on the basis of a required volume load of 8000 ml crystalloids during the first 24 hours and a standard deviation of 500 ml. A power of 80% and a significance level of 0.05 for a two sample t-test suggested that it would be sufficient to have three patients in each group if HH reduced the required volume by 30% to 5600 ml. Due to lower power in non-parametric tests, a higher number was chosen. The unconscious patients, as well as the neuroradiologist, were blinded to the treatment. The two treatment groups were descriptively compared at baseline. Fluid load, urine output and fluid balance were compared using an exact Mann-Whitney test. Mixed effects models were used for group comparisons of repeated measurements of variables [21]. Time from baseline was entered as a categorical covariate, as well as any differences in developments in the two groups, and there were assumed to be no group differences at baseline. The nlme package in R (R Foundation for Statistical Computing, Vienna, Austria) was used for linear mixed effects models; SPSS version 15.0 (SPSS Inc., Chicago, IL, USA) was used for other statistical analyses, and SPSS Sample Power for power calculation. Numbers were presented as mean (standard error), or median (low-high). A p-value <0.05 was considered significant. For categorical covariates with more than two categories, both overall p-values for the variable and p-values for individual contrasts are reported.

## Results

### Patients and outcome

**Figure 1:**
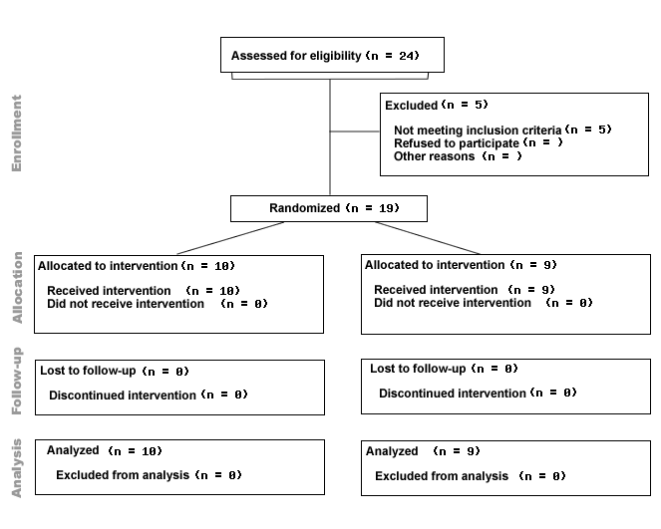
**CONSORT flowchart**.

Twenty-four patients were randomised. Five were excluded due to lack of witnessed arrest, inclusion in another study, probable respiratory cause of the cardiac arrest, and age >80 years (Fig 1). Ten patients (two female) were randomised to HH, and nine (one female) to the control fluid regimen. The initial heart rhythms and baseline characteristics are presented in Table 3. There were no substantial differences between the groups as regards the aetiology of the arrest. The first temperature recorded at the hospital was 34.5 (1.4) °C. Survival after one year was 79%, with no significant difference between the groups (Table 3).

**Table 3:** Prehospital data.

	HH	Control	Total
Number	10	9	19
Age (yrs)	60 (48-74)	60 (22-75)	
BMI (kg/m^2^)	26.2 (22.1-34.1)	26.2 (21.6-35.1)	
CA-CPR (min)	1 (0-4)	2 (1-9)	
CA-EMS (min)	8.5 (3.0-15.0)	7.0 (5.0-12.0)	
Ventricular fibrillation	8	8	
Ventricular tachycardia	1	0	
Asystole	1	1	
No of shocks	5 (1-11)	3 (1-16)	
Adrenaline (mg)	3 (0-15)	1 (0-10)	
CA-ROSC (min)	23 (5-40)	17 (10-39)	
Intra-Aortic-Balloon-Pump	3	0	
Survivors	8/10	7/9	15/19

Presented as median (range).

CA-CPR- time from cardiac arrest until cardiopulmonary resuscitation was started

CA-EMS- time from cardiac arrest until emergency medical staff was present

CA-ROSC- time from cardiac arrest until return of spontaneous circulation.

### Fluid

During the first 24 hours in the hospital, the HH group required significantly less fluid than the control group to meet the treatment goals. Fluid calculations are presented in Table 4. The HH group received 6.02 ml/kg (4.63 - 7.69) of HH during the first 24 hours.

**Table 4:** Fluid calculations after 24 hours.

	HH	Control	**p-value**^**a**^
Volume (ml/24 hrs)	4750 (3150-9075)	8010 (5515 - 12908)	0.019
Volume (ml/kg/hr)	2.67 (1.54 - 4.55)	4.00 (3.06 - 6.58)	0.004
Diuresis (ml/kg/hr)	0.97 (0.44 - 2.16)	1.43 (0.63 - 2.36)	0.24
Balance (ml/kg/hr)	+ 1.06 (0.20 - 4.11)	+2.27 (0.71 - 5.36)	0.040

Presented as median (range).

a) Exact Mann-Whitney test

### Oedema

**Figure 2:**
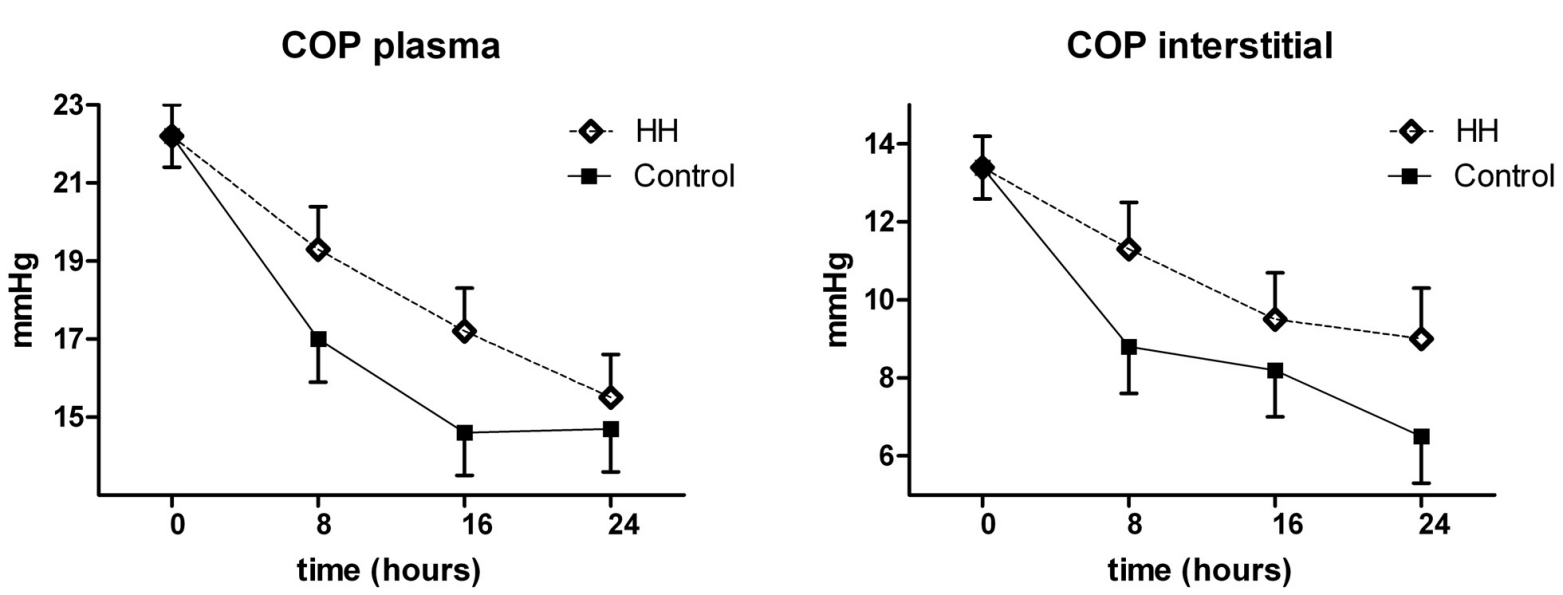
**Colloid osmotic pressure during cooling.** Mixed effects model with mean and standard error. a) Colloid osmotic pressure in plasma. Mixed effects model with mean and standard error. Overall p < 0.001. Changes 24 vs. 0 hours. p < 0.001 (both groups). b) Colloid osmotic pressure in interstitial tissue. Mixed effects model with mean and standard error. Overall p < 0.001. Changes 24 vs. 0 hours. p = 0.001/p < 0.001 (HH/Control).

COP in plasma showed a significant decline in both groups (Fig. 2a). The reduction was more rapid in the control than in the HH group, but the nadir levels were the same in both groups. The corresponding levels of interstitial COP showed the same pattern (Fig. 2b). The drop in COP was significant at all times, except for the HH group at eight hours. The planned MRI at 0, 24 and 96 hours was performed on seven patients. MRI was performed at 0 and 96 hours on two patients, and on one patient at 96 hours. The ten patients were equally distributed between the two groups. Divergence from the plan was due to technical problems. MRI did not reveal vasogenic cerebral oedema in any of these patients.

### Hemodynamics

**Figure 3:**
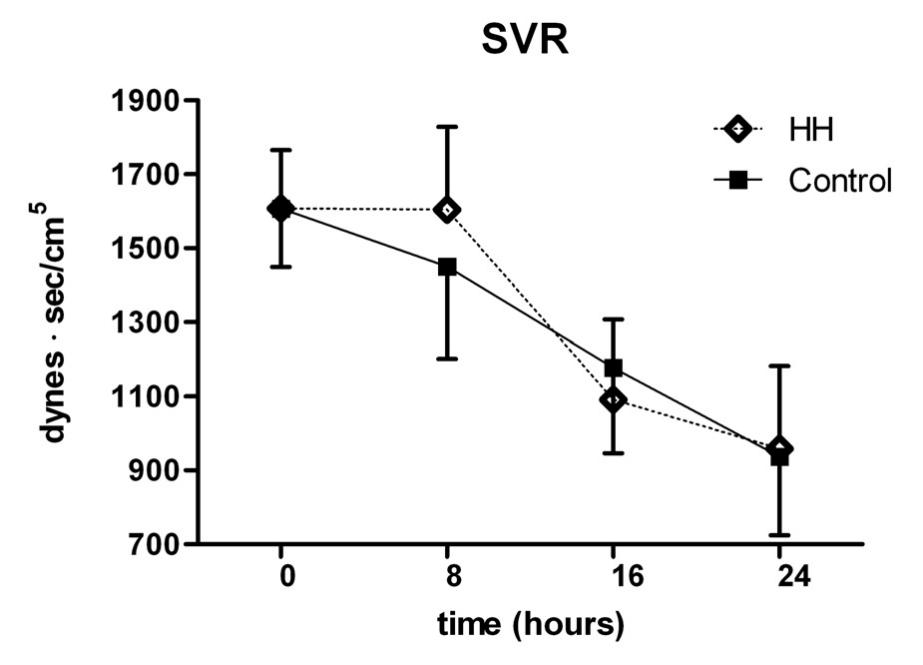
**Systemic vascular resistance during cooling**. Mixed effects model with mean and standard error. Overall p = 0.014. Changes 24 vs. 0 hours. p = 0.008/p = 0.005 (HH/Control).

**Figure 4:**
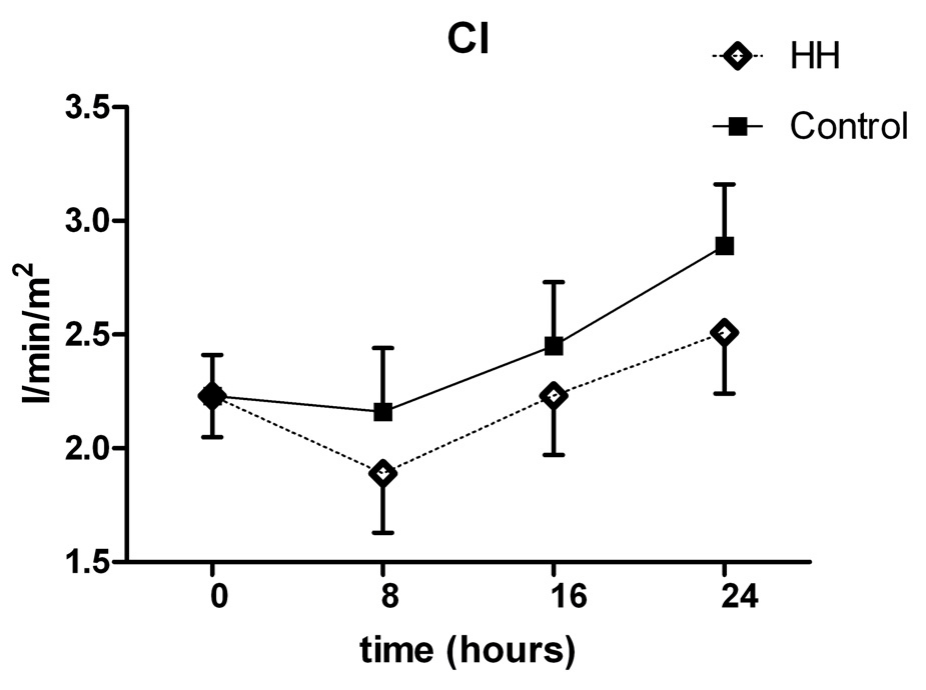
**Cardiac index**. Mixed effects model with mean and standard error. Overall p = 0.044. Changes 24 vs. 0 hours. p = 0.31/p = 0.019 (HH/Control).

**Figure 5:**
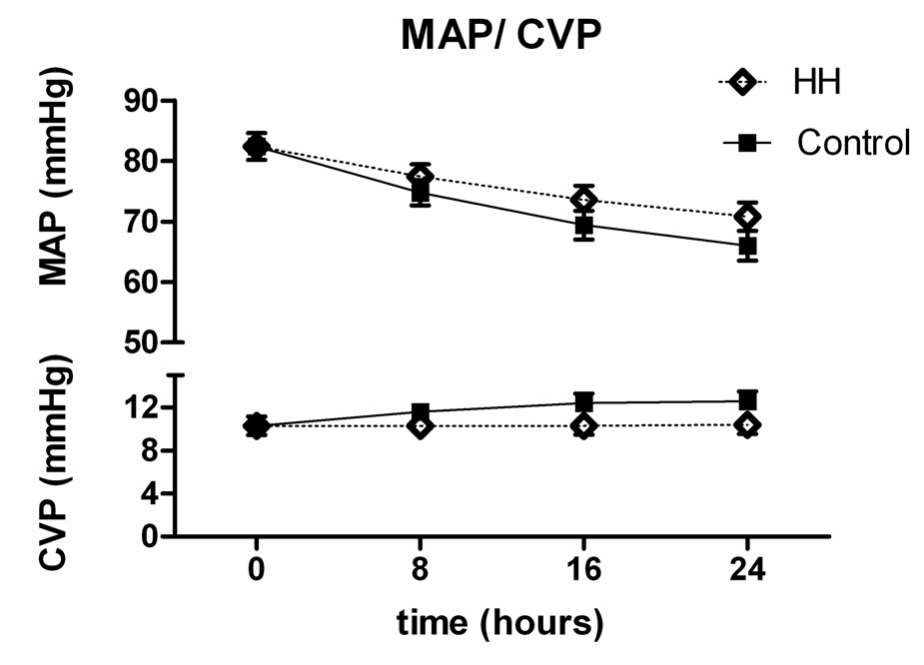
**Mean arterial pressure and central venous pressure (estimates and standard errors based on mixed effects models)**. Difference between slopes of curves at 0 hours, p = 0.20/0.12, and difference between curvatures p = 0.34/0.25 (MAP/CVP).

SVR dropped significantly in both groups (Fig. 3). The cardiac index (CI) was 2.2 l/min/m^2 ^(0.2) on admission to the MICU. At 24 hours, before rewarming, the CI was higher in both groups and significantly higher in the control group (Fig. 4). MAP and CVP did not differ significantly between groups (Fig. 5). There were no differences in dose and type of vasopressors between the groups. All patients needed vasopressors, primarily dopamine in accordance with the MICU guidelines.

### Laboratory data/adverse effects

All laboratory data are listed in Table 5. Serum osmolality differed significantly, with an increase in the HH group and a decrease in the control group (p < 0.001). Serum sodium and chloride increased in both groups. Two patients who received HH later developed renal failure.

**Table 5:** Laboratory: Calculated mean using mixed effects model at 0, 8, 16 and 24 hours after admission to the MICU.

Time (hrs)		**0**^**a**^	8	16	24	**p-value**^**b**^	**p-value**^**c**^	Overall p-value
Na^+^	HH	139	148	153	151	<0.001	<0.001	<0.001
mmol/l	Control	139	141	141	142	0.001		
Cl^-^	HH	100	116	125	121	<0.001	<0.001	<0.001
mmol/l	Control	100	107	109	110	<0.001		
K^+^	HH	4.1	3.8	3.9	3.9	0.542	0.798	0.869
mmol/l	Control	4.1	4.2	4.0	3.8	0.343		
Ca^2+^	HH	2.29	2.07	2.03	2.01	<0.001	0.689	<0.001
mmol/l	Control	2.29	2.14	2.01	2.04	<0.001		
Hb	HH	15.1	13.8	13.0	12.1	<0.001	0.158	<0.001
g/dl	Control	15.1	14.6	13.8	13.0	<0.001		
Hematocrit	HH	0.45	0.41	0.39	0.36	<0.001	0.183	<0.001
	Control	0.45	0.44	0.40	0.39	<0.001		
pH	HH	7.28	7.29	7.31	7.33	0.098	0.253	0.043
	Control	7.28	7.35	7.34	7.37	0.002		
Osmolality	HH	311	320	319	318	0.015	<0.001	<0.001
mosm/kg	Control	311	302	299	299	<0.001		

a Estimates based on mixed effects model, with no baseline differences assumed.

b Contrast from baseline at 24 hours

c Contrast between groups at 24 hours

## Discussion

We studied fluid requirements and oedema formation in survivors of OHCA in a prospective, randomised design. The HH patients received significantly less fluid than the control patients (4750 ml vs. 8010 ml, p = 0.019). Both groups had a significant drop in SVR, and demonstrated increased extravasation through the drop in COP. The extravasation did not show as vasogenic brain oedema.

The strength of the study lies in its design and the multiple determination of leakage. The weakness of our design is that the treating physicians were not blinded. This could have caused a tendency to replace fluid with vasopressors. However, there were no differences between the groups regarding the use of these drugs. Furthermore, as sedation can cause vasodilatation, the use of sedation may influence the use of fluid and vasopressors. The lowest doses of sedation were used in all patients to achieve MAAS 0-1. The number of patients in our study is not sufficient to determine whether fluid load can affect neurological outcome/survival.

A large cohort study recently reported on the challenging aspects of therapeutic hypothermia [22]. In spite of a positive fluid balance, many patients appear to be hypovolemic and have high fluid requirements [2]. Our reported fluid balance is slightly higher than the balance reported by Sunde and colleagues, who found a positive balance of 3455 ml (1594) during 24 hours with similar treatment goals [3]. Laurent and colleagues used 3500-6500 ml during the first 24 hours to maintain an adequate filling pressure in normothermic cardiac arrest patients [1]. Whether reduced fluid load is of benefit to these patients remains unknown.

To our knowledge, there are no papers describing repetitive MRI in the initial treatment of OHCA patients. Järnum and colleagues performed MRI on 20 cardiac arrest patients who remained unconscious 72 hours after normothermia [23]. They found hypoxic-ischemic cerebral oedema in two patients during neuropathological examination post mortem. None of the patients in our study had a vasogenic cerebral oedema on the MRI, which indicated an intact blood-brain barrier. Animal studies have shown that asphyxia is more likely to cause a disrupted blood-brain barrier [24-26]. The lack of vasogenic oedema may be the result of cardiac origin of the arrest, the fact that arrests were witnessed and short time before initiation of CPR.

We found reduced fluid leakage to the interstitial space in the HH patients compared with controls. Maintenance of intravascular COP is one important factor in determining fluid flux across the capillary membrane. The decline in COP in plasma was probably due to hemodilution, which is also reflected in a reduction in haemoglobin and erythrocyte volume fraction.

The reduction in COP in interstitial fluid is probably caused by the escape of fluid with a lower COP through the capillaries. Since we observed a simultaneous reduction in COP both in plasma and interstitial fluid, the increased extravasation cannot be explained by the differences in the COP gradient between the groups. However, the change may be attributed instead to capillary leakage, which has also been demonstrated in several animal studies [5,27,28]. This is supported by Nordmark et al., who found a decreased intravascular volume during hypothermia after cardiac arrest [2]. COP is important in capillary fluid exchange, but is a minor component of the total osmotic pressure. The significant difference between the groups regarding serum osmolality may partly explain the observed differences in fluid loads. This emphasises the importance of also taking the total osmotic pressure into consideration when choosing i.v. fluid. Sodium concentration in the HH group differed significantly from the controls after 24 hours and reflected the content of sodium in the HH solution. This may influence fluid shifts and lead to osmotic dehydration, with shrinkage of cells and the prevention of endothelial oedema [29].

Both groups demonstrated a comparable and significant reduction in SVR, suggesting a similarity between septic and post-cardiac arrest patients [30]. As hypovolemia leads to an increased SVR, our finding may reflect a volume 'overload' [31]. Hypothermia and infusion of vasopressors should induce vasoconstriction and centralise circulation. However, intravenous fluid and inflammation counteract vasoconstriction [32], and the overall result was a significant decline in SVR in both the study and the control group, also observed by Laurent et al. [1].

Small volume resuscitation with hypertonic saline during CPR is described as feasible and safe [29], and, in a study of critically ill ICU patients, HH was infused without negative effects on renal function [33]. The VISEP study [34] showed impaired renal function in sepsis patients resuscitated with hydroxyethyl starch 200/0.5, and there have been discussions concerning the safety of these solutions in critically ill patients. Two of our patients who received HH developed renal failure, one due to arterial embolism, while the other developed failure weeks later. We consider the kidney failure in these two patients to be unrelated to HH; its contribution cannot be excluded, however.

Despite lower body temperature, CI was higher at 24 hours than on admission to the MICU. The increase was significant in the control group. Laurent and collaborators made the same observation when they monitored more than 160 OHCA patients with pulmonary artery catheter [1]. The improvement in CI in our study represents adequate fluid load and reduced stunning of the heart. In a recent study, Jacobshagen et al. also demonstrated an improved ventricular function over time in patients after cardiac arrest [35].

The clinical implication of the present study is that post-cardiac arrest patients can be liberally infused with crystalloids during the first 24 hours without cerebral oedema resulting. They also have a high fluid requirement, which is partly because of increased extravasation, measured by means of colloid osmotic pressures, systemic vascular resistance and fluid calculations. Both fluid regimens stabilise hemodynamics. The reduced fluid load achieved by the application of HH should be further investigated in cardiac arrest caused by asphyxia, where a disrupted blood-brain barrier is more likely. The lack of vasogenic brain oedema in these patients is encouraging. This supports a liberal use of crystalloids, especially due to an increased need for intravascular volume and the possible side effects of colloids. Furthermore, the impact on neurological outcome and survival should be examined.

## Conclusions

Post-cardiac arrest patients have high fluid requirements during therapeutic hypothermia, probably due to increased extravasation. The use of HH reduced the fluid requirement significantly. However, the lack of brain oedema in both groups suggests no superior fluid regimen. Cardiac index was significantly improved in the group treated with crystalloids. Although we do not associate HH with the renal failures that developed, caution should be taken when using hypertonic starch solutions in these patients.

## Competing interests

The authors declare that they have no competing interests.

## Authors' contributions

BEH participated in the design of the study, the application for official approvals and the collection and interpretation of data. JKH, ABG participated in the design of the study and in collection and interpretation of data. JL, RF participated in the design of the study and collection of data. SMH, EML participated in the collection and interpretation of data. TWL participated in the design of the study and statistical analysis of the data. All authors read and approved the final manuscript.
